# NADPH accumulation is responsible for apoptosis in breast cancer cells induced by fatty acid synthase inhibition

**DOI:** 10.18632/oncotarget.15936

**Published:** 2017-03-06

**Authors:** Yanfen Cui, Pan Xing, Yuanyuan Wang, Miao Liu, Li Qiu, Guoguang Ying, Binghui Li

**Affiliations:** ^1^ Laboratory of Cancer Cell Biology, National Clinical Research Center for Cancer, Tianjin Key Laboratory of Cancer Prevention and Therapy, Tianjin Medical University Cancer Institute and Hospital, Tianjin 300060, China

**Keywords:** fatty acid synthase, apoptosis, redox homeostasis, breast cancer

## Abstract

Fatty acid synthase (FAS), as a key enzyme involved in *de novo* lipogenesis, is highly expressed in many cancers. FAS inhibition induces cell death *in vivo* and *in vitro*, rendering FAS as an attractive target for cancer therapy, but the defined mechanism is still not well understood. Herein, we confirmed that FAS was highly expressed in breast cancers and FAS inhibition by its inhibitors or knockdown induced apoptosis in breast cancer cells. Our results showed that a significantly high level of reactive oxygen species was induced but not responsible for apoptosis in breast cancer cells by FAS inhibition. Instead, NADPH accumulation resulting from FAS inhibition was found to stimulate NADPH oxidase to generate reactive oxygen species and highly associated with apoptosis induction. Suppression of NADPH oxidase almost totally blocked reactive oxygen species generation while significantly potentiated the *in vitro* and *in vivo* killing of breast cancers by FAS inhibition. Taken together, these data suggest that FAS plays a critical role in maintaining cellular redox homeostasis and its inhibition leads to NADPH accumulation-mediated apoptosis. Our finding may provide new insights into cancer metabolism and aid in designing effective anticancer treatments.

## INTRODUCTION

In contrast to normal tissues that mainly utilize exogenetic lipids, cancers cells tend to undergo active *de novo* lipogenesis [1, 2]. Fatty acid synthase (FAS), as a key enzyme involved in *de novo* lipogenesis, catalyzes the synthesis of palmitate from the substrates of acetyl-CoA, malonyl-CoA and NADPH. Increased levels of FAS have emerged as a typical phenotype of most cancers, including breast cancer, colorectal cancer, ovarian cancer, and so on [3–5]. Furthermore, a high level of FAS is reported to be associated with poor prognosis and anticancer drug resistance in cancer patients [6, 7].

FAS is a potential target for cancer therapy, and several small-molecule FAS inhibitors, such as cerulenin and orlistat, are extensively studied. Cerulenin is isolated from Cephalosporium caerulens and contains an epoxy group that can irreversibly react with FAS [8]. Similarly, orlistat is also an irreversible inhibitor forming a covalent adduct with the active serine of thioesterase domain in FAS [9]. These FAS inhibitors induces apoptosis in cancer cells both *in vivo* and *in vitro* and have been utilized as potential treatments for cancers [3, 10, 11]. FAS inhibition-induced apoptosis could be mediated by endoplasmic reticulum stress, increased reactive oxygen species (ROS) or accumulated ceramide [12–15]. However, the detailed mechanism by which FAS inhibition induces apoptosis still remains to be explored.

To access the mechanism of apoptosis in breast cancer cells induced by FAS inhibition, we detect the level of NADPH, a substrate of FAS, after treatment with FAS inhibitors or knockdown in the current study. Our results reveal that FAS inhibition perturbs the homeostasis of NADPH/NADP^+^, which results in the generation of ROS and is responsible for FAS inhibition-induced apoptosis.

## RESULTS

### FAS is hyper-expressed in breast cancer tissues and related to cancer recurrence

**Figure 1:**
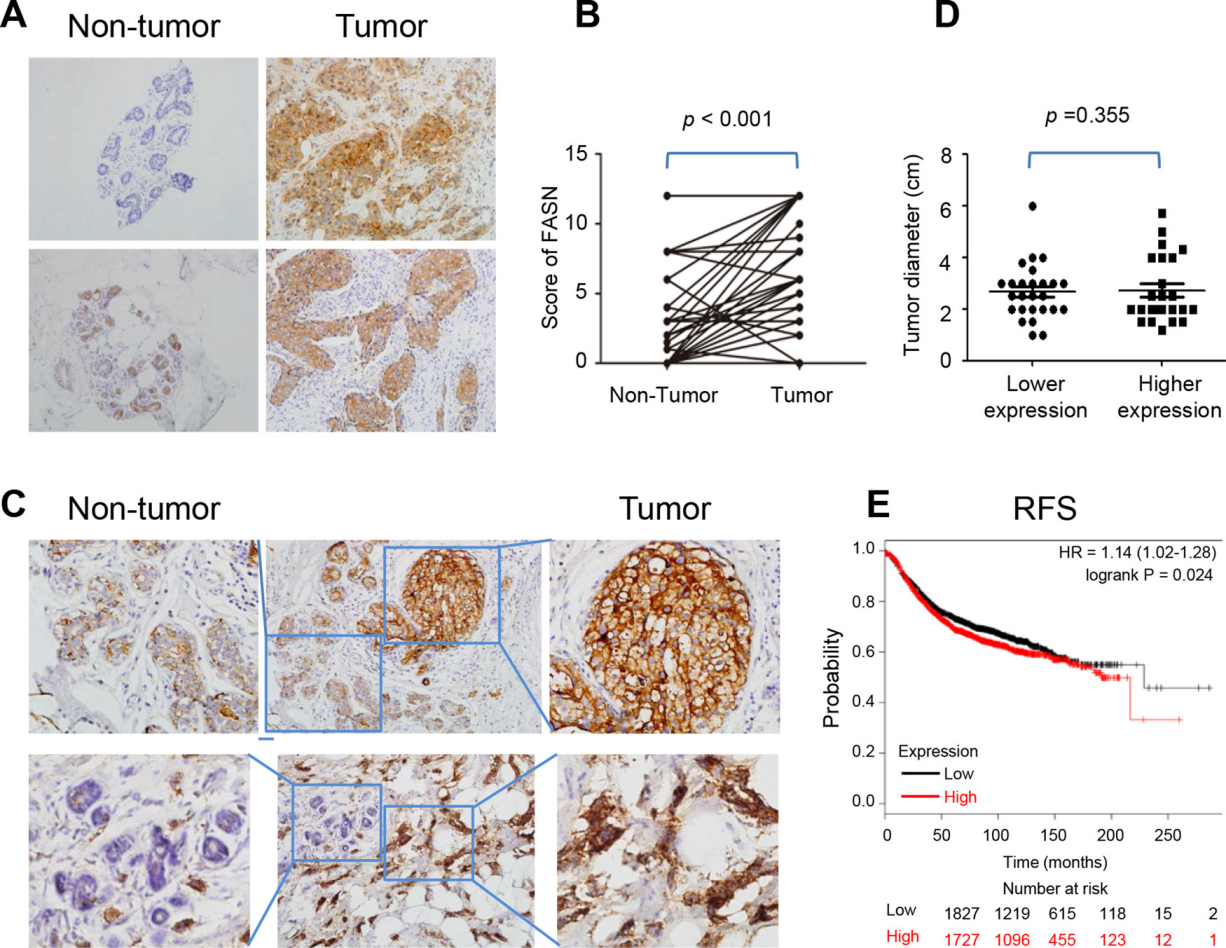
Expression of FAS in breast cancer tissues (**A**) The present IHC pictures of FAS in non-tumor breast and breast cancer tissues. (**B**) The scores of FAS in 50 paired non-tumor breast and breast cancer tissues. (**C**) The expression level of FAS in non-tumor breast was lower than breast cancer tissues in the same microscopic vision. (**D**) The relationship between tumor diameter and FAS expression level. FAS lower expression: score 0–4; FAS higher expression: score 5–12. (**E**) The higher FAS abundance correlated with a poor recurrence-free survival based on microarray data of 3554 breast patients in the website: www.kmplot.com.

To investigate FAS expression in breast cancers, immunohistochemistry (IHC) was applied to compare the expression level of FAS in breast cancers with that in non-tumor breast tissues in 50 patients. The results showed that cancer tissues expressed a much higher level of FAS than adjacent non-tumor breast tissues (Figure 1A and 1B), which was in agreement with previous studies [16, 17]. This was further confirmed by parallel results in the same samples (Figure 1C). In addition, we analyzed the correlations of FAS expression with clinicopathological variables of cancer, including age, tumor diameter, clinical stage, lymphatic metastasis, distant metastasis and recurrence status in these breast cancer patients (Table 1). There was no significant relationship between the diameter of cancers and FAS expression level (Figure 1D), suggesting that a higher level of FAS expression does not additionally promote cell proliferation.

**Table 1:** Correlation between FAS expression and clinicopathologic characteristics of human breast cancers

Variables	*n*	FAS expression		χ^2^	*P*
		Low	High		
Age (years)					
< 50	27	8	19	0.004	1.000
≥ 50	23	7	16		
Clinical stage					
Early (stage I)	23	5	18	1.384	0.355
Advanced(stage II–IV)	27	10	17		
Tumor Diameter					
≤ 2 cm	23	5	18	1.384	0.355
> 2 cm	27	10	17		
Lymph nodeMetastases					
No	30	6	24	3.571	0.114
Yes	20	6	11		
Distant Metastases					
No	48	15	33	0.893	1.000
Yes	2	0	2		
Recurrence					
No	48	15	33	0.893	1.000
Yes	2	0	2		

To investigate the prognosis of FAS for breast cancer, we plotted the Kaplan-Meier survival curves for FAS in www.kmplot.com, and found that the higher FAS abundance correlated with a poor recurrence-free survival (RFS) via microarray data from *n* = 3554 breast patients (HR = 1.14, *P* = 0.024) (Figure 1E). By contrast, no statistical significance for overall survival (OS) or distance metastasis free survival (DMFS) was found (Supplementary Figure 1). It suggests that patients with higher FAS expression are prone to recurrence. Although there was no statistical significant relationship between FAS level and cancer recurrence in 50 patients in the current study due to the limited case number, 2 of 35 patients with high FAS expression (score = 12) while none of patients with low FAS expression had cancer recurrence (Table 1).

### Inhibiting FAS by inhibitors or shFAS induces apoptosis in breast cancer cells

**Figure 2:**
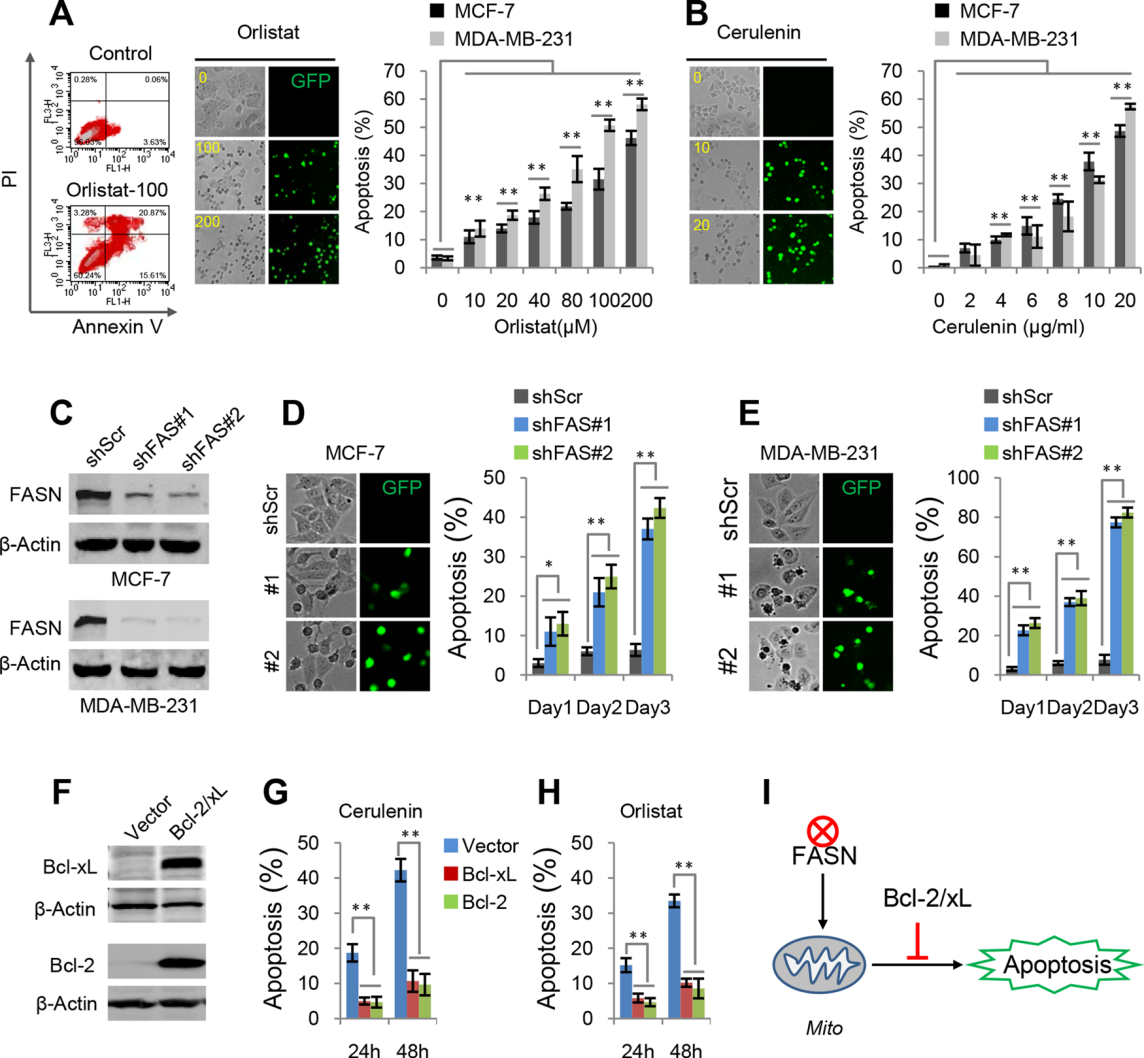
Apoptosis induced by FAS inhibitors and shFAS (**A**) Orlistat-induced apoptosis in MCF-7 and MDA-MB-231 cells. Apoptotic cells were quantified by Annexin V staining or green fluorescence from the apoptosis biosensor, GC3AI. The similar results upon two methods were obtained. (**B**) Cerulenin-induced apoptosis in MCF-7 and MDA-MB-231 cells. Apoptosis was detected by the apoptosis biosensor GC3AI. (**C**) Western blots for FAS knockdown in MCF-7 and MDA-MB-231 cells. (**D** and **E**) Apoptosis in MCF-7 and MDA-MB-231 cells induced by shFASs. (**F**) Western blots for Bcl-2 and Bcl-xL over-expression in MCF-7 cells. (**G** and **H**) Effects of over-expressed Bcl-2 and Bcl-xL on cerulenin- or orlistat-induced apoptosis in MCF-7 cells. 10 μM of cerulenin and 100 μg/ml of orlistat were used. (**I**) A model for FAS inhibition-induced mitochondria-dependent apoptosis. Error bar indicates ± SE (*n* = 3). *p < 0.05; **p < 0.01 (*t*-test).

FAS inhibitors, cerulenin and orlistat, have been shown to induce apoptosis *in vivo* and *in vitro*, but their specific mechanism is still not well understood. Here, our recently developed apoptosis biosensor, GC3AI whose fluorescence was activated by caspase-3-like protease during apoptosis, was used to real-time monitor apoptosis [18]. Our results showed that cerulenin and orlistat induced concentration-dependent apoptosis in breast cancer MCF-7 and MDA-MB-231 cells (Figure 2A and 2B). These results were consistent with those obtained from annexin V-staining flow data (Figure 2A).

To further interrogate the role of FAS inhibition in apoptosis, shRNAs were used to deplete FAS expression in breast cancer cell lines MCF7 and MDA-MB-231 (Figure 2C). FAS depletion by shFASs also induced significant apoptosis in both breast cancer cell lines in three days (Figure 2D and 2E). However, a fraction of shFAS cells survived FAS depletion-induced cell death and appeared to adapt to FAS knockdown, here referred to as the adapted shFAS cells. Analysis of these cells showed that the FAS protein level was still significantly suppressed although not as dramatically as that in the initial shFAS cells (Supplementary Figure 2A). Furthermore, we found that the adapted shFAS cells showed the similar proliferation rate but the reduced colony formation ability in the conditions of 2D and 3D cell culture, compared with the control cells (Supplementary Figure 2B–2D). These results suggest that serious inhibition of FAS could induce apoptosis while mildly inhibiting FAS also suppress the colony formation of breast cancer cells.

Over-expression of Bcl-2 and Bcl-xL (Figure 2F), anti-apoptotic mitochondrial proteins, significantly blocked cerulenin- and orlistat-induced apoptosis in MCF7 cells (Figure 2G and 2H), suggesting that FAS inhibition induces mitochondria-dependent apoptosis (Figure 2I).

### ROS generation is induced by FAS inhibition but it is not responsible for apoptosis

**Figure 3:**
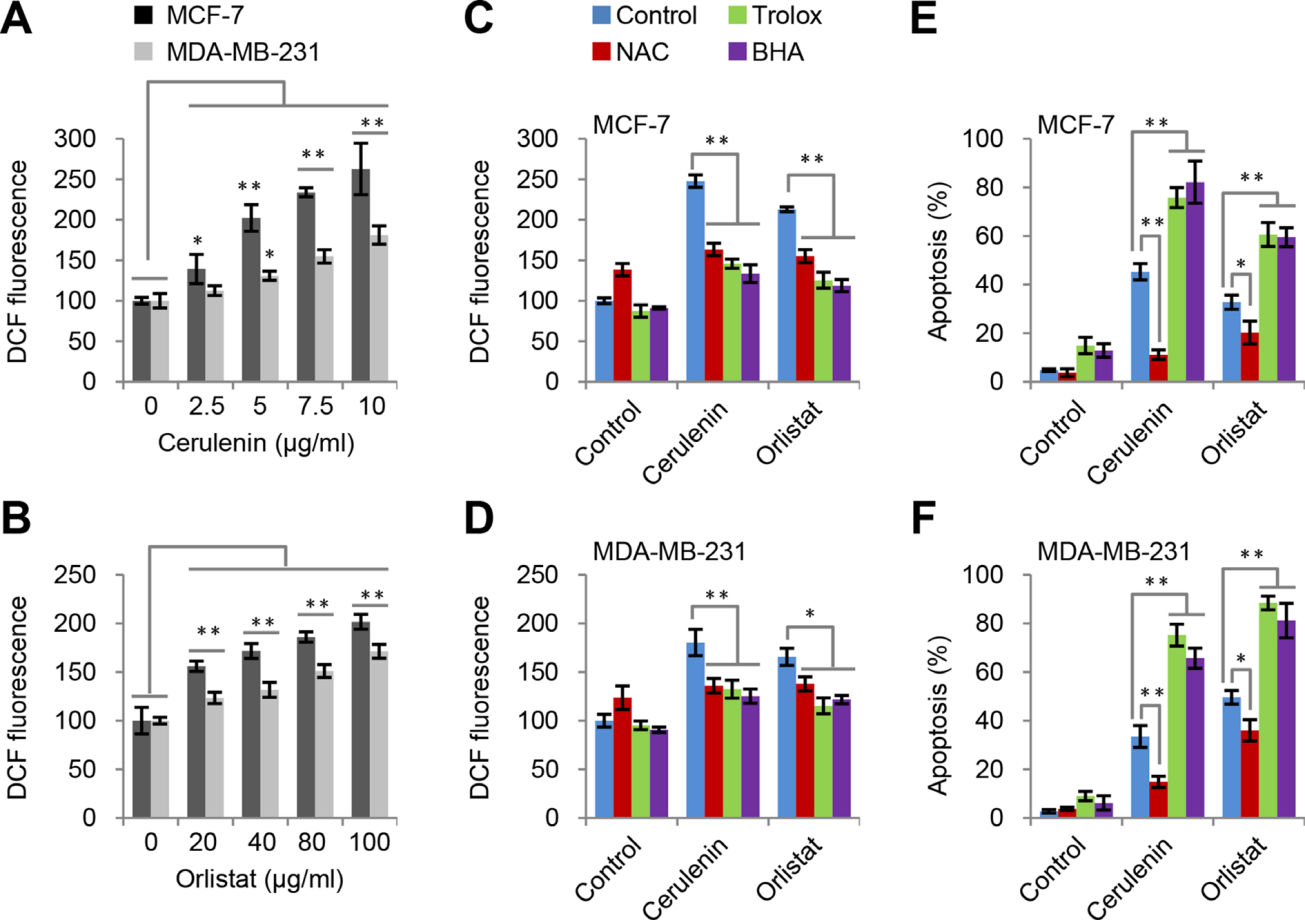
ROS is induced by FAS inhibition but not responsible for apoptosis (**A** and **B**) Cerulenin and orlistat induced ROS generation in MCF-7 and MDA-MB-231 cells. ROS levels were measured after cells were treated with FAS inhibitors as indicated for 2 h. (**C** and **D**) Effects of trolox, BHA and NAC on ROS generation in MCF7 and MDA-MB-231 cells induced by cerulenin and orlistat. ROS levels were measured after cells were treated with 10 μM of cerulenin or 100 μg/ml of orlistat in combination with 2 mM of trolox, 10 mM of NAC or 100 μM of BHA for 2 h. (**E** and **F**) Effects of trolox, BHA and NAC on apoptosis in MCF-7 and MDA-MB-231 cells induced by cerulenin and orlistat. Apoptosis was measured after cells were treated with 10 μM of cerulenin or 100 μg/ml of orlistat for 48 h. Error bar indicates ± SE (*n* = 3).*p < 0.05; **p < 0.01 (*t*-test).

Previous reports suggested that FAS inhibition could induce ROS generation [19, 20]. Here, we also observed that cerulenin and orlistat led to concentration-dependent and time-dependent increase in ROS levels in both MCF-7 and MDA-MB-231 cell lines (Figure 3A and 3B, Supplementary Figure 3A).

Next we determined if inhibiting ROS prevented apoptosis induced by FAS inhibitors. N-acetylcysteine (NAC) [21], an aminothiol and synthetic precursor of intracellular glutathione, was used to inhibit ROS. The results showed that the high levels of ROS induced by cerulenin and orlistat were reduced by NAC treatment in both MCF7 and MDA-MB-231 cell lines (Figure 3C and 3D). Meanwhile, apoptosis induced by FAS inhibitors was also significantly reduced (Figure 3E and 3F). To further confirm these observations, another two strong ROS scavengers, butylated hydroxyanisole (BHA) and trolox, were used to wipe off ROS. Intriguingly, the results showed that both BHA and trolox significantly decreased the level of ROS in both MCF7 and MDA-MB-231 cells (Figure 3C and 3D), but failed to inhibit and actually exacerbated apoptosis induced by FAS inhibitors (Figure 3E and 3F). These paradoxical results on ROS suggest that ROS is likely not the real cause to induce FAS inhibition-mediated apoptosis.

### NADPH accumulations is responsible for apoptosis

**Figure 4:**
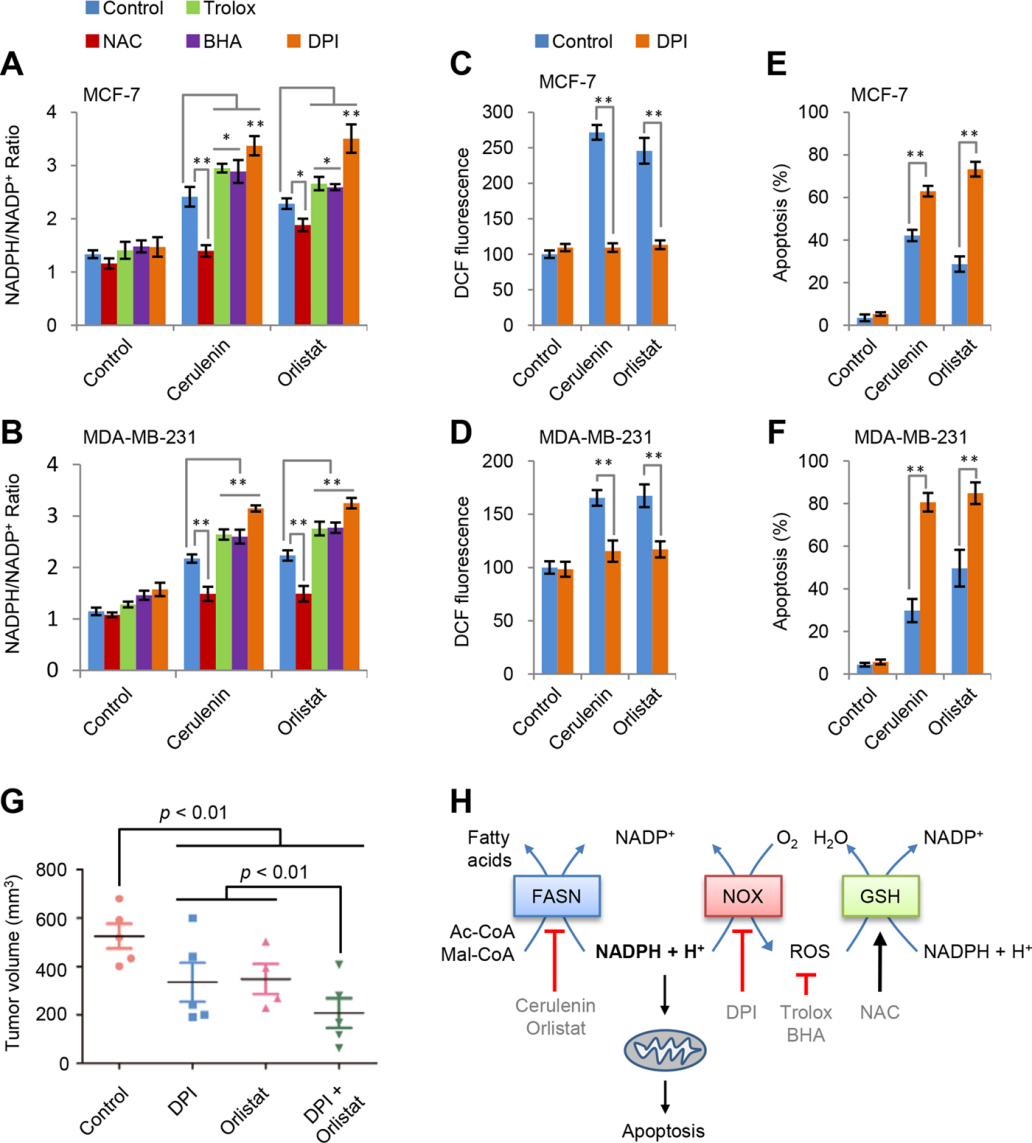
NADPH accumulation is responsible for apoptosis (**A** and **B**) Effects of trolox, BHA, NAC and DPI on NADPH/NADP^+^ ratio in MCF-7 and MDA-MB-231 cells induced by cerulenin or orlistat. NADPH/NADP^+^ was measured after cells were treated with 10 μM of cerulenin or 100 μg/ml of orlistat in combination with 2 mM of trolox, 10 mM of NAC, 100 μM of BHA or 5 μM of DPI for 2 h. (**C** and **D**) Effects of DPI on ROS generation in MCF-7 and MDA-MB-231 cells induced by cerulenin and orlistat. ROS levels were measured after cells were treated with 10 μM of cerulenin or 100 μg/ml of orlistat in combination with 5μM of DPI for 2 h. (**E** and **F**) Effects of DPI on apoptosis in MCF-7 and MDA-MB-231 cells induced by cerulenin and orlistat. Apoptosis was measured after cells were treated with 10 μM of cerulenin or 100 μg/ml of orlistat in combination with 5 μM of DPI for 48 h. Error bar indicates ± SE (*n* = 3). *p < 0.05; **p < 0.01 (*t*-test). (**G**) DPI and orlistat synergistically suppressed tumor growth of MDA-MB-231 cells in a xenograft model. (**H**) A model to show the mechanism underlying FAS inhibition-induced apoptosis.

ROS usually results from redox stress. Considering that redox-associated NADPH is a substrate of FAS, FAS inhibition could lead to NADPH accumulation. Our results confirmed that cerulenin and orlistat induced concentration-dependent NADPH accumulation in both MCF-7 and MDA-MB-231 cells (Supplementary Figure 3B and 3C). Furthermore, we measured the effects of NAC, BHA and trolox on NADPH accumulation. As shown in Figure 4A and 4B, NAC suppressed while BHA and trolox promoted the increased ratio of NADPH/NADP^+^ in both MCF-7 and MDA-MB-231 cells by cerulenin and orlistat. It appears that the variation tendency of NADPH/NADP^+^ ratio is consistent with apoptosis induced by FAS inhibitors and ROS scavengers.

Besides mitochondria, cytosolic NADPH oxidases (NOX) were the major ROS source [22]. To test if NOX was involved in FAS inhibition-induced ROS generation, diphenyleneiodonium (DPI), an inhibitor of NOX [23], was used. The results showed that DPI almost completely blocked FAS inhibitors-induced ROS generation (Figure 4C and 4D) but increased NADPH/NADP^+^ ratio in MCF7 and MDA-MB-231 cells (Figure 4A and 4B). Although DPI decreased ROS, it did not prevent but instead promoted apoptosis in both MCF7 and MDA-MB-231 cells induced by FAS inhibition (Figure 4E and 4F). These data further confirmed that the real reason for apoptosis induced by FAS inhibitors was NADPH accumulation but not increased ROS levels.

In view of the promotion of FAS inhibitors-induced cell death by DPI, we tested their actions on *in vivo* growth of MDA-MB-231 cells in nude mice. When the tumor volumes reached to around 50 mm^3^, all mice were divided into four treatment groups, control (treated with vehicles), DPI (treated with DPI), orlistat (treated with orlistat) and DPI + orlistat (treated with both DPI and orlistat). The DPI and orlistat combination treatment showed significantly inhibitory effects on tumor growth as compared to the control and single treatment groups (Supplementary Figure 4A and 4B). DPI or orlistat alone also inhibited tumor growth, and their combination treatment showed a synergetically inhibitory effect on tumor development (Figure 4G).

## DISCUSSION

Our data suggest that FAS inhibition-induced apoptosis most likely results from NADPH accumulation. As illustrated in Figure 4H, FAS inhibition leads to the accumulation of NADPH, one of its substrates. Subsequently, the accumulated NADPH activates NOX to generate ROS that can actually help to restore redox homeostasis of NADPH/NADP^+^ through the glutathione system. Different antioxidants exert diverse effects on ROS and NADPH/NADP^+^ homeostasis depending on their mechanisms. NAC, as the precursor of glutathione, removes ROS mainly via amplifying glutathione system [21, 24], and thus decreases both ROS level and NADPH/NADP^+^ ratio (Figures 3A, 3B, 4A and 4B). By contrast, BHA and trolox directly scavenge ROS so that they lead to an increase in NADPH/NADP^+^ ratio (Figure 4A and 4B). The NOX inhibitor DPI blocks NOX-mediated ROS generation but significantly potentiates FAS inhibition-induced NADPH accumulation (Figure 4A–4D). As a result, NAC prevents while BHA, trolox and DPI promote FAS inhibition-induced apoptosis in breast cancer cells (Figures 3E, 3F, 4E and 4F). Taken together, these results are consistent with the interpretation that NADPH accumulation not ROS generation is responsible for FAS inhibition-induced mitochondria-dependent apoptosis in breast cancer cells. However, a strong ROS stress signal resulting from FAS inhibition may also directly trigger apoptosis in some types of cells in some conditions [13, 14, 19, 25].

Unlike normal tissue cells, cancer cells have active *de novo* lipogenesis [26]. As a key enzyme involved in lipogensis, FAS is highly expressed in many cancers including breast cancers and is supposed to support cell proliferation [27]. However, our results showed that FAS expression in breast cancer tissues was not positively correlated to the diameters of cancers. Therefore, the higher level of FAS expression appears not to additionally promote cell proliferation, but it is associated with cancer recurrence. These results suggest FAS could exert some important roles in cell survival in addition to cell proliferation. As revealed in our current study, FAS probably functions as a critical modulator in maintaining redox homeostasis of cancer cells. Indeed, FAS-mediated fatty acid synthesis consumes a large amount of NADPH [28]. To maintain redox homeostasis by dissipating NADPH, the synthesized fatty acids may be oxidized in mitochondria, if they are over the requirements for cell proliferation. This may explain why sometimes cancer cells have active fatty acid synthesis and oxidation at the same time [27, 29].

FAS recently emerges as a potential target for cancers, and thus its inhibitors are widely tested in cancer treatments [27]. Here, based on the mechanism underlying FAS inhibition-induced apoptosis in breast cancer cells elucidated in our current study, we found a NOX inhibitor DPI can synergistically increase the killing ability of FAS inhibitor orlistat to breast cancers in a xenograft model. Therefore, our finding may be therapeutically helpful to the application of FAS inhibitors.

## MATERIALS AND METHODS

### Patients and tissue samples

Breast cancer tissues and adjacent non-tumor tissues were obtained from 50 patients in Department of Pathology, Tianjin Medical University Cancer Institute and Hospital in 2015. All tissue sections were examined by specialists, and histopathological diagnoses were made using the World Health Organization criteria. The classification of cancer stage and grade was according to the International Federation of Gynecologyand Obstetrics (FIGO, 2009). Clinicopathological data were collected including age, histology type, ascites, metastases status and tumor grade. All patients’ characteristics were summarized in Table 1.

### General reagents

Reagents cerulenin, orlistat, DPI, BHA, trolox and NAC were all obtained from sigma. The NADP^+^/NADPH Quantitation Colorimetric Kit (K347-100) was used to detect the NADP^+^/NADPH ratio obtained from BioVision.

### Cell culture

Breast cancer cell lines MCF-7 and MDA-MB-231 were obtained from ATCC (Rockville, MD, USA), and cells were cultured in DMEM supplemented with 10% FBS (Biological Industries, Israel) inside an incubator containing 5% CO_2_ at 37°C.

### Immunohistochemistry

FAS expression was detected by immuno histochemistry. Fifty clinical specimens of primary breast cancers and matching non-tumor tissues were analyzed for expression of FAS. The sections were incubated with FAS antibody (1:50 dilution) for overnight at 4°C, then incubated with PV6001 (Zhongshan Goldbridge Biotechnology CO., Ltd., Beijing, China) for 30 min at 37°C and stained with DAB for 1 to 2 min. A semi-quantitative method and blind scoring were utilized by pathologist. As described [30], the extent of expression score was assessed on a scale of 0 to 4 (no positive cells = 0, 1–25% = 1, 26%–50% = 2, 51–75% = 3, >50% = 3), and the intensity score was also measured on a scale of 0 to 3 (negative = 0, weak = 1, moderate = 2, strong = 3). Multiplication of the values for intensity and extent of expression provided a score for immunoreactivity. For statistical analysis, samples with scores of 0–4 were considered to be low expression, while scores of 5–12 were considered to be high expression.

### Western immunoblotting

The lysis buffer (20 mM of Tris-HCl, pH 7.5, 150 mM of NaCl, 1 mM of EDTA, 1% Triton X-100, 2.5 mM of sodium pyrophosphate, 1 mM of β-glycer-ophosphate, 1 mM of sodium vanadate, 1 mg/mL of leupeptin, and 1 mM of phenylmethyl-sulfonylfluoride) was used to obtain total protein, whose concentration was measured by Bradford method. 20 μg of protein was separated on a 10% of SDS-PAGE gel and blotted onto a PVDF membrane. 5% of fat-free milk blocks the membrane, which was then incubated with primary antibody for 1h and secondary antibody for 1h at room temperature [31]. The antibodies used in this study as following: FAS (Santa Cruz, Cat#sc-48357), Bcl-2 (Abcam, Cat#ab692), Bcl-xL (Abcam, Cat#ab77571). β-Actin (Santa Cruz, Cat#sc-1616) was used as an internal control. The secondary antibodies for western blot were obtained from Li-Cor, and Li-Cor Odyssey image reader (Li-Cor, USA) was used to western detection.

### Determination of apoptosis

For the apoptosis assay by FACS analysis, 25 × 10^4^ cells were seeded into each well of the 6-well plates. Apoptosis assays were carried out based on the instruction from the Annexin V Apoptosis Kit (BD Biosciences). Briefly, cells were collected and washed twice with binding buffer containing 10 mM of HEPES, pH 7.4, 140 mM of NaCl, 2.5 mM of CaCl_2_, and then resuspended at a concentration of 1 × 10^6^ cells/ml in binding buffer. One hundred microliters of the cell suspension was mixed with 5 μl of Annexin V-FITC and 10 μl of propidium iodide (50 μg/ml stock) and incubated at room temperature for 15 min. Four hundred microliters of binding buffer was added to each assay after the incubation and apoptotic cells were determined using a FACScan (BD Biosciences). Total Annexin V-positive cells were used to determine the level of apoptosis.

For the apoptosis assay by GC3AI biosensor [18], cells expressing GC3AI were grown in twelve–well plates. After the desired treatments, cells were then rapidly imaged with an EVOS^®^ FL digital inverted fluorescence microscope with 10× objective lens. The filter sets for imaging were: GFP: Ex470/22, Em525/50; PI (RFP): Ex531/40, Em593/40. GFP-positive cells were counted as apoptosis. Five random areas in each well were imaged, and each area contained more than two hundreds of cells. Cell counting was performed with ImageJ software (1.47) by analyzing these pictures.

### Intracellular ROS measurement

2′,7′-Dichlorodihydrofluorescein diacetate (H_2_DCFDA) (Invitrogen) was used to measure intracellular ROS, which was oxidized to fluorescent 2′,7′-dichlorofluorescein (DCF) in the presence of ROS. Cells washing with PBS two times after treatment with reagents were incubated with 20 μM of H_2_DCFDA at 37°C for 30 min. To remove excess probe, the cells were washed with PBS again, and the fluorescence intensity was excited at 495 nm and measured at 527 nm emission wavelengths. Fluorescence intensity was calculated with ImageJ software (1.47) by analyzing these pictures.

### Lentivirus generation and infection

Human Bcl-xL and Bcl-2 cDNA and apoptosis biosensor GC3AI cDNA were subcloned into the lentiviral expressing vector pCDH-CMV-EF1-puro (System Biosciences). shRNAs against FAS, CCCTGAGATCCCAGCGCTGTT and CATGGAGCGTATCTGTGAGAA, and a scramble sequence CCTAAGGTTAAGTCGCCCTCG were subcloned into the pLKO.1 lentivirus expressing vector. Viral packaging was done according to the previously described protocol [29]. Briefly, expressing plasmid pCDH-2/Bcl-xL or pLKO.1-shFAS, pCMV-dR8.91 and pCMV-VSV-G were co-transfected into 293 T cells using the calcium phosphate method at 20:10:10 μg (for a 10-cm dish). The transfection medium containing calcium phosphate and plasmid mixture was replaced with fresh complete medium after incubation for 5 h. Media containing virus was collected 48 h after transfection and then concentrated using 20% sucrose buffer at 20,000 g for 4 h. The virus pellet was re-dissolved in the proper amount of complete growth medium and stocked at −80°C. Cancer cells were infected with the viruses at the titer of 100% infection in the presence of Polybrene (10 μg/ml) for 48 h, and were treated as desired.

### Animal tumor model and treatment groups

2 × 10^6^ MDA-MB-231 cells were suspended in 100 μl of PBS and injected into the flank of 6 week-old nu/nu mice. When tumors volume reached around 50 mm^3^,the mice were randomly divided into four groups (*n* = 5 per group): (a) control (200 μl of PBS thrice weekly by intraperitoneal injection); (b) DPI (1 mg/kg once per day by intraperitoneal injection) [32]; (c) orlistat (orlistat was extracted from capsules, and prepared according to methods reported by HuiYen Chuang at el [33], 240 mg/kg once per day by intraperitoneal injection); (d) DPI and orlistat combination (240 mg/kg of orlistat once per day by intraperitoneal injection, and 1mg/kg of DPI once per day by intraperitoneal injection). Therapy was continued for 20 days, and tumor volumes were detected every four days. As described, tumor volumes were determined using the formula: volume (mm^3^) = *a* × *b*^2^ × 0.5 (*a* is the long diameter and *b* is the short diameter) [34]. On day 20 after treatment, all mice were sacrificed, and tumors were weighted and photos. All of the data are represented as means ± SE.

### Statistics

Data are given as means ± SE. Statistical analyses were performed using unpaired, two-tailed Student's *t*-test for comparison between two groups. Asterisks in the figure indicated statistical significances (*p < 0.05; **p < 0.01).

## SUPPLEMENTARY MATERIALS FIGURES AND TABLES


