# Supplementary material for: NADPH accumulation is responsible for apoptosis in breast cancer cells induced by fatty acid synthase inhibition
Source: Oncotarget. 2017 Mar 6;8(20):32576–85. doi: 10.18632/oncotarget.15936 (PMC5464810; doi:10.18632/oncotarget.15936)
Supplement: Supplementary file 1 [file oncotarget-08-32576-s001.pdf]

NADPH accumulation is responsible for apoptosis in breast cancer cells induced by fatty acid synthase inhibition

Supplementary Materials

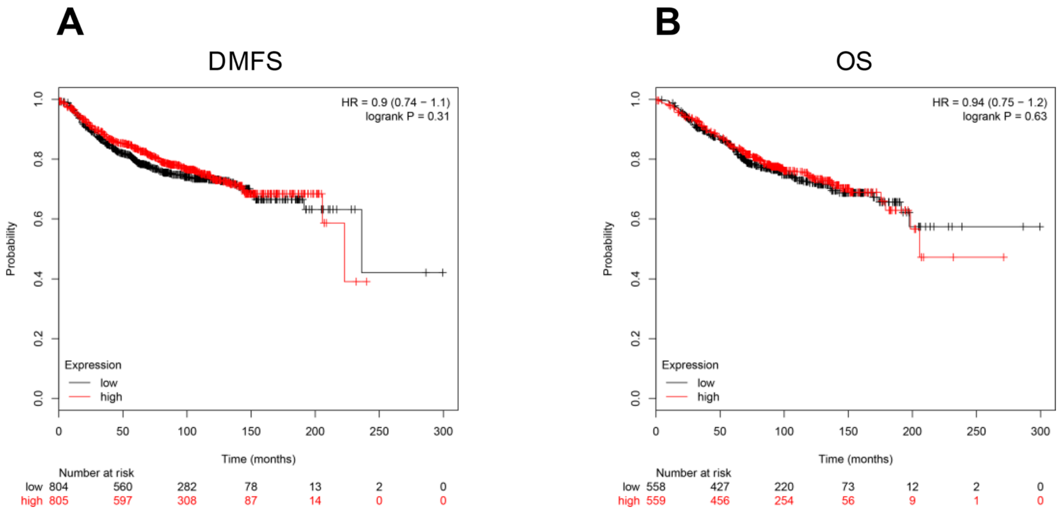

Supplementary Figure 1: Effects of FAS on breast cancer patients' overall survival (OS) and distance metastasis free survival (DMFS). The plotted data in the Kaplan-Meier survival curves for FAS were obtained in the website: www.kmplot.com.

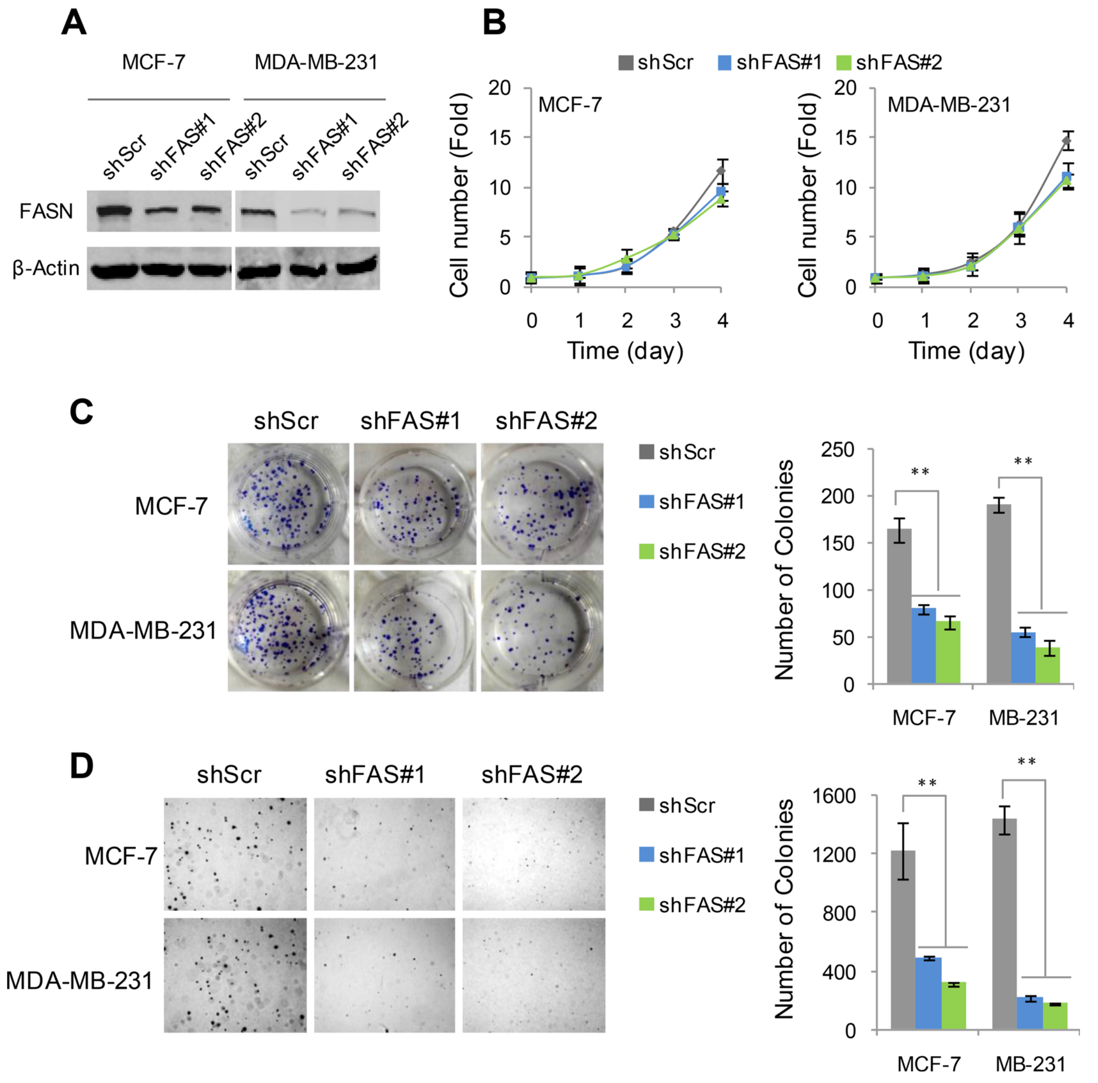

Supplementary Figure 2: Effects of FAS knockdown on growth of breast cancer cells. (**A**) Western blots of FAS knockdown effect in MCF-7 and MDA-MB-231 cells. Cells, termed the adapted shFAS cells, survived shFAS-induced apoptosis after passages. (**B**) Growth rate of the adapted shFAS cancer cells. (**C**) The colony formation ability of the adapted shFAS cancer cells in 2D cell culture. (**D**) The colony formation ability of the adapted shFAS cancer cells in 3D soft agar cell culture. Error bar indicates ± SE ($n = 3$).*$p < 0.05$; **$p < 0.01$ (t-test).

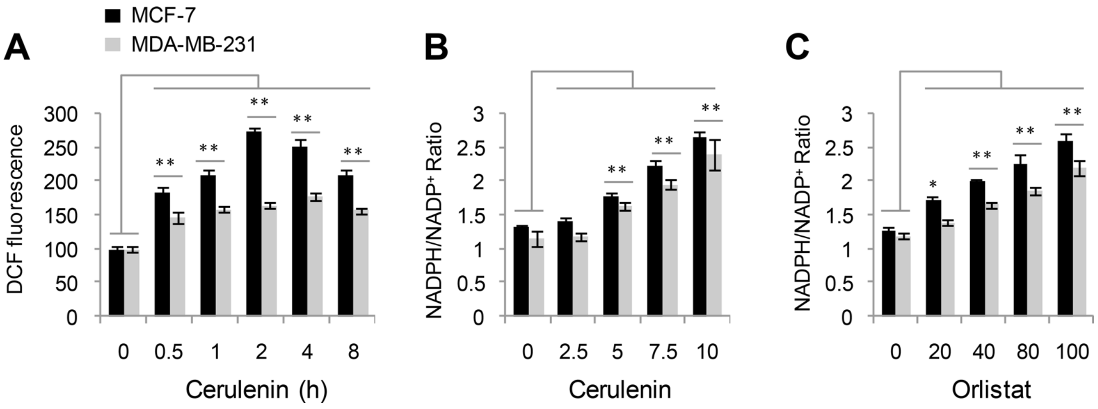

Supplementary Figure 3: Effects of FAS inhibitors on cellular ROS and NADPH/NADP$^+$. (**A**) Cerulenin induced time-dependent ROS generation in MCF-7 and MDA-MB-231 cells. ROS levels were measured after cells were treated with 10 μM of cerulenin or 100 μg/ml of orlistat for different times as indicated. (**B** and **C**) Cerulenin and orlistat induced concentration-dependent increase in NADPH/NAD^{P+} ratio in MCF-7 and MDA-MB-231 cells. NADPH/NAD^{P+} ratios were measured after cells were treated with different concentrations of cerulenin or orlistat for 2 h. Error bar indicates ± SE (n = 3).*$p < 0.05$; **$p < 0.01$ (t-test).

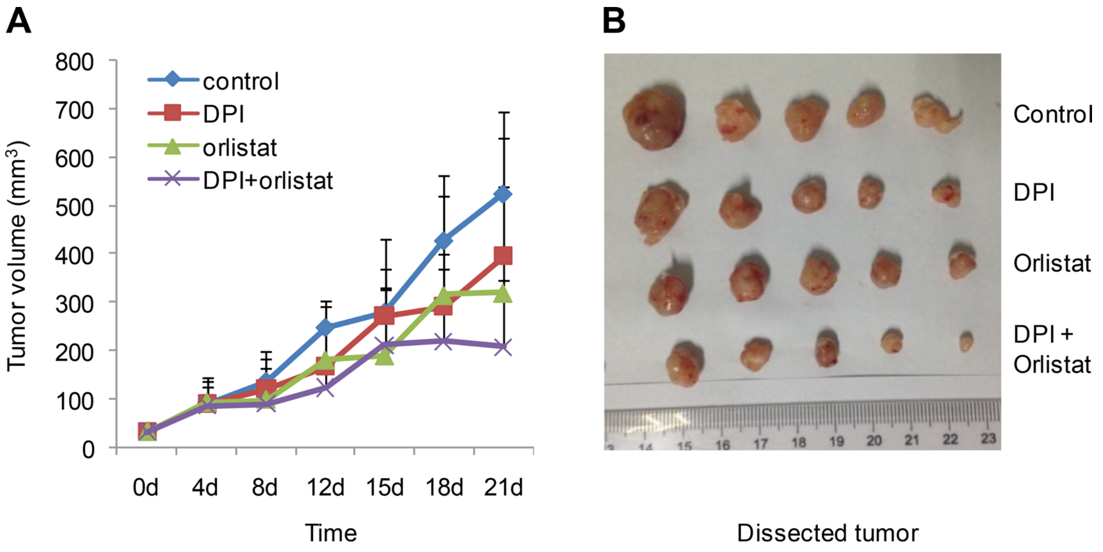

Supplementary Figure 4: Effects of orlistat and DPI on the *in vivo* killing of breast cancers. (**A**) Tumor growth curves of MDA-MB-231 cells in a xenograft model with different treatment as indicated. (**B**) The images of dissected tumor on day 20 after treatment drugs.
